# Applications of Mesenchymal Stem Cells in Liver Fibrosis: Novel Strategies, Mechanisms, and Clinical Practice

**DOI:** 10.1155/2021/6546780

**Published:** 2021-08-10

**Authors:** Mengmei Zhu, Tianzhen Hua, Tao Ouyang, Huofu Qian, Bing Yu

**Affiliations:** ^1^Department of Cell Biology, Center for Stem Cell and Medicine, Naval Medical University (Second Military Medical University), Shanghai 200433, China; ^2^Department of Gastroenterology, The Second People's Hospital of Taizhou, China

## Abstract

Liver fibrosis is a common result of most chronic liver diseases, and advanced fibrosis often leads to cirrhosis. Currently, there is no effective treatment for liver cirrhosis except liver transplantation. Therefore, it is important to carry out antifibrosis treatment to reverse liver damage in the early stage of liver fibrosis. Mesenchymal stem cells (MSCs) are the most widely used stem cells in the field of regenerative medicine. The preclinical and clinical research results of MSCs in the treatment of liver fibrosis and cirrhosis show that MSC administration is a promising treatment for liver fibrosis and cirrhosis. MSCs reverse liver fibrosis and increase liver function mainly through differentiation into hepatocytes, immune regulation, secretion of cytokines and other nutritional factors, reduction of hepatocyte apoptosis, and promotion of hepatocyte regeneration. Recently, many studies provided a variety of new methods and strategies to improve the effect of MSCs in the treatment of liver fibrosis. In this review, we summarized the current effective methods and strategies and their potential mechanisms of MSCs in the treatment of liver fibrosis, as well as the current research progress in clinical practice. We expect to achieve complete reversal of liver injury with MSC-based therapy in the future.

## 1. Introduction

Liver fibrosis is a fibrotic and inflammatory process caused by chronic liver injury. During liver fibrosis, the continued accumulation of extracellular matrix (ECM), which is rich in collagen I and III, leads to scar deposition [1]. The most common pathogeneses are viral hepatitis infections (hepatitis B and C viral infections (HCV/HBV)), alcoholism, nonalcoholic fatty liver disease (NAFLD)/nonalcoholic steatohepatitis (NASH), and autoimmune hepatitis [2–6]. Although their pathogenesis is different, their common endpoint is the development of liver cirrhosis. Due to the lack of effective treatment, reversible liver fibrosis may develop into cirrhosis [7]. Liver cirrhosis has steadily increased morbidity and mortality in high-income countries and has become a major health problem worldwide. Although liver transplantation offers hope to patients with end-stage liver disease, it is still an expensive and complex procedure with significant side effects [8].

Mesenchymal stem cells (MSCs) are pluripotent stem cells that have received a broad focus in differentiation, transplantation, and immune response in various diseases [9, 10]. MSCs can be isolated from a variety of tissues and are suitable for experimental and possible clinical applications [11]. Many studies have shown that MSCs can provide effective treatments for animal models of liver fibrosis and cirrhosis, and a number of clinical trials have been conducted. In this review, we discuss the therapeutic potential of MSCs in liver fibrosis and new strategies to improve their antifibrotic capacity. We summarize the current effective methods and potential mechanisms of MSCs treatment of liver fibrosis and discuss the current clinical trial process.

## 2. Source of MSCs

MSCs are the most widely used stem cells that have the unique ability to self-renew and differentiate into many different cell types. For the existing sources of mesenchymal stem cells, the two main issues regarding cell therapy are the cell donor and the location from which the cells are isolated.

### 2.1. MSCs are Isolated from Different Locations

MSCs are stromal cells that can be easily isolated from a variety of tissue sources, including bone marrow, placenta, umbilical cord, amniotic fluid, adipose tissue, dental pulp, breast milk, and synovium [12]. MSCs can be culture-expanded and will not be rejected after transplantation. At present, many studies have demonstrated that bone marrow- [13, 14], umbilical cord- [15, 16], and fat- [17, 18] derived mesenchymal stem cells can inhibit liver fibrosis in preclinical animal models, suggesting their potential application in the treatment of liver fibrosis. Furthermore, Park et al. firstly demonstrated that tonsil-derived mesenchymal stem cells (T-MSCs) can differentiate into hepatocyte-like cells and ameliorate liver fibrosis via autophagy activation and downregulation of TGF-*β* [19]. The transplantation of human amnion-derived mesenchymal stem cells (hAMSCs) may provide significant improvement in rat liver fibrosis models by inhibiting the activation of Kupffer cells and hepatic stellate cells [20, 21]. Moreover, a preclinical study shows that the infusion of human amniotic stem cells effectively decreases portal pressure by ameliorating liver microcirculation [22]. These findings suggest that MSCs may serve as new potential approaches to treat liver fibrosis and can be used as a new source of stem cell therapy for liver disease.

### 2.2. Advantages of Allogeneic MSCs for Therapeutic Applications

The cell donor can be the same as the cell recipient (autologous) or different from the cell recipient (allogeneic). In recent clinical trials of liver fibrosis and cirrhosis, the trend of MSC therapy seems to have shifted from the administration of autologous cells to allogeneic cells. The choice of autologous therapy is ideal because they ensure major histocompatibility and are unlikely to cause immunological rejection. However, autologous therapy still has some potential limitations, and it may be difficult to obtain a sufficient number of healthy active MSCs from patients [23]. The advantages of using allogeneic compared to autologous MSCs have been fully demonstrated, of which the most notable thing is to obtain cells from healthy donors and proliferate to required number in vitro. Another commonly touted advantage of allogeneic MSCs is their low immunogenicity. Due to their immunomodulatory properties and low immunogenicity, they have become a promising approach to treat graft-versus-host disease (GVHD) and autoimmune disease [24]. Lohan et al. introduced the causes of the immunosuppressive properties and low immunogenicity of allogeneic MSCs through changes in the expression of immunogenic markers on the cell surface and changes in the secretion of immunosuppressive molecules [25]. Although there may be some reasons compared with other allogeneic cell types, some results show that allogeneic MSCs can indeed induce a strong immune response in the body, which may lead to serious consequences [26]. Although various studies have reached inconsistent results on the treatment of allogeneic mesenchymal stem cells, allogeneic mesenchymal stem cells are still promising choices in immunosuppression and tissue repair therapy.

## 3. The Applications of MSCs in the Treatment of Liver Fibrosis

**Figure 1:**
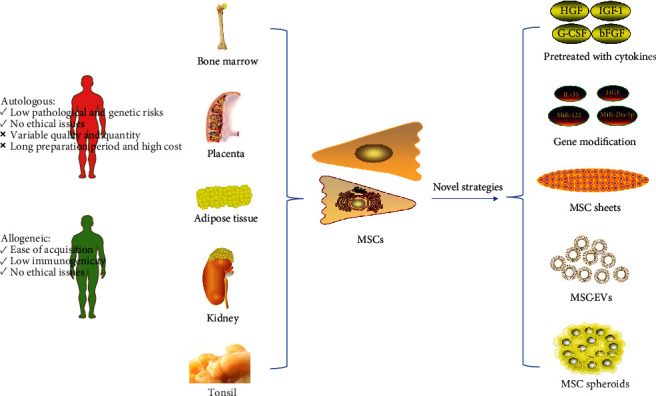
Sources of mesenchymal stem cells and novel strategies for the applications of MSCs in liver fibrosis. MSCs can be obtained from various sources, including bone marrow, placenta, adipose tissue, kidney, and the tonsil. Autologous and allogeneic MSCs have different therapeutic characteristics.

Cell-based therapy using MSCs has been proven to be beneficial to alleviate liver fibrosis in some basic and clinical studies. To further strengthen the therapeutic effect of MSCs in liver fibrosis, many MSC-based treatment methods for liver fibrosis have been explored and tested. Here, we summarized the proposed possible strategies to improve the antifibrotic ability of MSCs. The sources of MSCs and novel strategies for the applications of MSCs in liver fibrosis were summarized in Figure 1.

### 3.1. Pretreatments Enhance the Therapeutic Effects of MSCs in Liver Fibrosis

When cultured in vitro, the proliferative ability and activity of MSCs were often affected by culture media and additives (such as glucose level, growth factors, trace elements, lipids, and vitamins) as well as culture conditions and processes, including the oxygen concentration in the incubator, cell dissociating agent, and the density of the inoculated cells [27]. In addition, the regenerative capacity of MSCs was significantly decreased by the harsh microenvironment of injured organ, anoikis, and inflammation after transplantation in vivo, while pretreatment with growth factors, cytokines, chemical agents, hypoxia, inflammatory microenvironment, and gene modification can not only protect MSCs against injury but also improve the hepatogenic differentiation, homing capacity, survival, and paracrine effects of MSCs in vitro and in vivo, thus increasing the ability to attenuate liver injury [28].

In vitro data showed that MSCs pretreated with cytokines have better antifibrosis potential. The pretreatment of MSCs with melatonin (MT) has shown encouraging results in animal models of myocardial infarction, renal ischemia, and cerebral ischemia. Mortezaee et al. used this strategy for the treatment of CCl4-induced liver fibrosis [29]. The results showed that MT pretreatment was at play in improving the homing potential of BMMSCs and can better maintain the balance between matrix degradation and accumulation factors. Fiore et al. showed that insulin-like growth factor-I- (IGF-I-) pretreated MSCs are able to induce hepatic macrophages (hMø) to transform from profibrotic to a resolutive phenotype, as a key early event driving liver fibrosis amelioration [30]. The combination of granulocyte colony-stimulating factor (G-CSF) and MSCs will greatly improve the prognosis of patients with advanced liver disease treated with stem cells [31]. Pretreatment of adipose tissue-derived stromal cells (ADSCs) with basic fibroblast growth factor (bFGF) promoted the transdifferentiation of ADSCs into liver lineage cells in vitro, thereby reducing liver fibrosis in mice [32].

### 3.2. Gene Modification Enhances the Therapeutic Effects of MSCs in Liver Fibrosis

A series of genes and microRNAs with clear biological functions have been introduced into MSCs through viral or nonviral vectors to improve their differentiation, immune regulation, homing ability, and other repair-related abilities. Hepatocyte growth factor (HGF) is a potent mitogen for mature hepatocytes, which has been shown to play critical roles in liver regeneration and has been applied in gene therapy in cirrhosis and achieved a better therapeutic effect [33]. Hence, HGF were broadly used to modify MSCs to improve the therapeutic effects on liver fibrosis [34–36]. Kim et al. showed that cell therapy with genetically engineered human bone marrow MSCs, which were overexpressed HGF mediated by adenovirus, significantly promotes liver function and attenuates liver fibrosis than treatment with MSCs alone [34]. Seo et al. also showed that transplantation of human HGF-overexpressing human umbilical cord blood-derived MSCs (hHGF-HUCB-MSCs) in CCL4-induced rat liver fibrosis model has higher liver function improvement and lower collagen fiber deposition than treatment with unmodified HUCB-MSCs [35]. Moreover, the other genes, such as erythropoietin (EPO) [37], forkhead box A2 (Fox A2) [38, 39], FGF4 [40], FGF21 [41], IGF-1 [42]and IL-10 [43], modified MSCs also showed better therapeutic effect in the treatment of liver fibrosis.

MiR-122 is abundant in the adult liver and is a core player in liver biology and disease [44]. Lou et al. showed that miR-122 modified adipose tissue-derived MSCs (AMSC-122), which were constructed through lentivirus-mediated transfer of pre-miR-122, more effectively inhibited the proliferation of hepatic stellate cells (HSCs) and the maturation of collagen [45]. In the latest study, the secreted proteome released from AMSC-122 has higher antifibrotic and anti-inflammatory properties than the pure secreted proteome [46]. The research by Chen et al. reported that inhibition of miR-26a-5p can further improve the therapeutic effect of MSCs on liver cirrhosis through increasing the expression of HGF protein in MSCs [47].

In addition, gene-modified MSCs can also enhance the immunomodulation of MSCs, thus increasing the therapeutic effect of MSCs. For example, MSCs overexpressing IL-35 have higher immunosuppressive capabilities. IL-35-MSCs induce CD4 + T cells to produce IL-10, but have no effect on IFN-*γ* [48, 49]. Therefore, modifying some specific genes is a potential new strategy for treating liver fibrosis.

### 3.3. MSC Sheets Have Great Therapeutic Potential

Tissue engineering and/or regenerative medicine is the field of life science that uses engineering and biological principles to create new tissues and organs and promote the regeneration of damaged or diseased tissues and organs [50]. “Cell sheet engineering” is a unique scaffold-free tissue technology, which does not require trypsin digestion. Compared with tissue suspension injection or tissue engineering, the number of cells in the membrane is larger. In addition, transplanted cells can be efficiently delivered to the damaged site and fully maintain their viability at the targeted site [51, 52]. MSC sheets have been successfully applied to the repair of various tissues and organs including Achilles tendon [53], bone tendon [54], blood vessels [55], kidney [56], and heart [57]. In the study of antifibrosis, MSC sheets also have great potential. Itaba et al. combined cell sheet technology with a single small-molecule compound, IC-2, and the results showed that orthotopic transplantation of IC-2 engineered MSC sheets can significantly ameliorate liver fibrosis caused by long-term administration of CCl4 [58]. However, Fernández-Colino et al. analyzed cases of tissue engineering and regenerative medicine (TERM) interventions with adverse outcomes, suggesting that they may also trigger fibrosis [59]. How to improve the therapeutic potential of MSC sheets has become a question for many people. Chemical disruption (e.g., trypsin or collagenase enzyme treatment) deconstructs the extracellular matrix and intercellular proteins (via cell–cell and cell–ECM junctions). Nakao et al. used temperature responsive cell culture dishes (TRCD) cell sheet technology to harvest human umbilical cord mesenchymal stem cell (HUC-MSC) sheets which retain the typical structure of natural tissue-like interconnected cells, including ECM components and cell connections [60]. The results show that these proteins are kept intact in MSC cultures using cell sheet technology to enhance stem cell survival and its function in stem cell-based therapies. Chuah et al. believed that different combinations of matrix properties (including stiffness, roughness, and wettability) can affect MSC behaviors, such as adhesion, diffusion, and proliferation during cell sheet development [61]. In addition, Rahmi G and others proposed that attention should be paid to the fate of implanted cells in vivo, and multimodal imaging was used to track cell sheets and noninvasively follow-up of their regenerative characteristics [62].

### 3.4. The Application of MSC-Derived Extracellular Vesicles (MSC-EVs) in Liver Fibrosis

Extracellular vesicles (EVs) can be secreted by almost any type of cells and are found to be high levels in biological fluids. According to the size of the vesicles and the method of cell release, extracellular vesicles can be roughly divided into three subtypes: exosomes (30–130 nm), microvesicles (100–1000 nm), and apoptotic bodies (50–4000 nm) [63, 64]. Multiple studies have consistently demonstrated that extracellular vesicles transfer proteins, lipids, and RNA between various cell types, participate in important biological functions, and serve as a means of communication between cells [65, 66]. MSCs are considered to be the strongest cells that produce EVs. MSC-EVs play an important role in repairing bone damage, skin damage, and nerve damage, but the mechanism of action is not clear. A number of recent studies have investigated the therapeutic effect of MSC-EVs in animal models of liver disease.

Sabry et al. found that bone marrow-derived MSC (BM-MSC) microvesicles can promote the regression of rat liver fibrosis induced by CCL4 by reducing serum alanine aminotransferase (ALT), collagen-1*α* and IL-1*β, increasing* serum albumin levels and inactivating the TGF-*β*/Smad signaling pathway [67]. Another study investigated the role of hUCMSC-EVs in liver repair in *S. japonicum*-infected mice. They pointed out that by reducing type I/III collagen, the activation of hepatic stellate cells induced by TGF-*β*1 is suppressed, and liver damage of schistosomiasis is improved. At the same time, the levels of TNF-*α* and IL-1*β* decreased significantly, indicating that hUCMSC-EVs inhibited the inflammatory response [68]. Similarly, Jiang et al. found that hUCMSC-EVs have the potential of antioxidant and hepatoprotective and can reduce CCl4-induced liver damage by reducing apoptosis and oxidative stress [69]. Studies by Mardpour et al. showed that ES-MSC EVs increased the secretion of anti-inflammatory cytokines (examples: TGF-*β* and IL-10) and reduced IFN-*γ* level in TAA-induced chronic rat liver injury, showing immunoregulatory activity to ameliorate liver fibrosis [70]. In addition, Qiu et al. summarized the mechanism and role of miRNA transfer in mediating MSC-EVs in human disease models [63]. For example, in a mouse model of CCl4-induced liver fibrosis, MSC-EVs with miRNA-181-5p overexpression ameliorated liver fibrosis by autophagy activation [71]. Moreover, the identification of particular miRNAs at a given concentration within EVs circulating in the bloodstream would be used as molecular biomarkers for disease diagnosis and prognosis monitoring [72].

Therefore, for liver fibrosis and other liver injuries, MSC-EVs may be a promising alternative to MSC treatment. It should be noted that although the experimental results strongly suggest the therapeutic potential of MSC-EVs, there is still a lot of experimental work to be done before putting MSC-EVs into clinical application. However, MSC-EVs will not fully realize its potential in new cell-free therapy until the key questions such as the separation and purification of MSC-EVs, long-term storage, donor, and tissue source are solved.

### 3.5. The Application of MSC Spheroids in Liver Fibrosis

Three-dimensional (3D) cell culture technology is a new method of MSC culture in vitro. There are a variety of methods for 3D culture of MSCs, including microsphere culture, biopolymer scaffold culture, and 3D culture of hydrogel [73]. There are significant differences in cell phenotypes and biological activities between 3D cell culture and 2D monolayer cell culture. MSC microsphere culture is considered to be an optimal way to improve MSC cell therapy [74]. MSC microspheres have been shown to improve the stemness of MSCs, enhance anti-inflammatory effects, enhance angiogenesis, and promote tissue regeneration and repair [75].

MSC spheroids robustly enhance the therapeutic potential of MSCs in following ways. First, the 3D-cultured MSCs are evenly distributed in organs like the liver, heart, and kidney, enhancing the internal microcirculation of MSCs [76]. Second, pluripotent gene expression of MSC spheroids, such as OCT4, Nanog, SOX-2, and REX-1 is increased. The senescence of MSCs cultured in vitro is delayed, while multipotent differentiation and stemness of MSC spheroids are also enhanced [77]. Third, MSC spheroids play an anti-inflammatory role by secreting anti-inflammatory factors and regulating immune cells, such as inhibiting the activation of macrophages [78]. Last, MSCs cultured in 3D spheres had high expression of cytokines related to angiogenesis and tissue repair to enhance angiogenesis and tissue regeneration [79].

Specifically speaking, MSCs with various sources have confirmed their effects in managing liver fibrosis. Study showed that hUC-MSC spheroids could migrate to the injured liver more effectively compared with the hUC-MSCs in 2D culture. The hUC-MSCs could promote liver regeneration and repair in mice with liver injury [80]. Yoshiaki et al. utilized stem cells from human exfoliated deciduous teeth (SHED) to develop microhepatic tissues. SHED-converted hepatocyte-like cells (SHED-HLCs) form human 3D-spheriacal microhepatic tissues and exert the therapeutic effect by secreting bioactive products like hALB in mice, thus providing a new method in treating chronic liver fibrosis [81]. Another research indicates that 3D cultured human adipose-derived MSCs (AD-MSCs) were endowed with higher expression of antifibrotic factors like IGF-1, IL-6, and HGF and also better capability of protecting hepatocyte injury and apoptosis. Transplanted AD-MSCs are expected to reverse hepatic fibrosis and improve hepatic function [82].

The utilization of MSC spheroids was not all booming. Restrictions still occur in several aspects. Firstly, the transport of nutrients and oxygen as well as the waste in the 3D sphere are restricted by the size of sphere, causing relatively high mortality of cells in the center. Secondly, the culture system composed of MSCs and 3D scaffold biomaterials is transplanted concurrently; the immunogenicity and histocompatibility of the biomaterials should be fully considered. Finally, differences still occur between the constructed 3D culture system and the environment in vivo, and the interaction mechanism between cells and ECM remains to be elucidated [83–85].

Despite these restrictions, MSC spheroids show great application prospects in the field of regeneration. MCS multicellular spheres may replace single-liver cells as the cellular component of current artificial liver, providing novel strategies to obtain artificial liver with low immunogenicity and solving the problem of donor shortage in liver transplantation.

## 4. Mechanisms of MSC-Based Therapy in Liver Fibrosis

**Figure 2:**
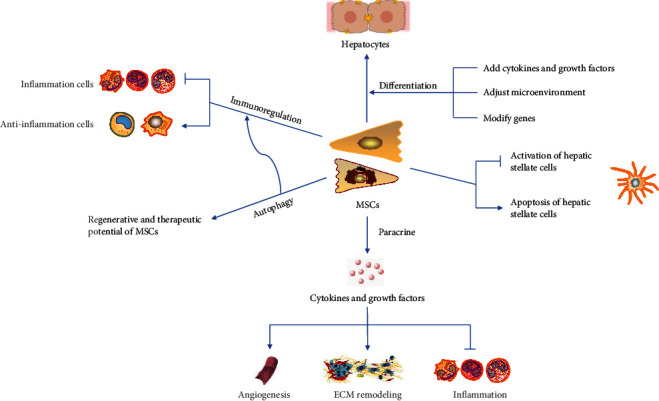
The potential mechanisms of MSC-based therapy in liver fibrosis. MSCs have various effects including the differentiation of hepatocytes and the regulation of inflammation response. Additionally, MSCs affect the regenerative process through autophagy and paracrine effects.

MSC administration is a promising therapeutic approach that can promote liver regeneration and repair liver fibrosis through the migration of cells into liver, hepatogenic differentiation, paracrine mechanisms, autophagy, and immunoregulation. Many articles have introduced the potential mechanisms of MSCs in treating liver fibrosis. Here, we supplement the latest research progress. The potential mechanisms of MSC-based therapy in liver fibrosis were shown in Figure 2.

### 4.1. MSCs Had the Potential for Differentiating into Hepatocyte-Like Cells

MSCs have plasticity and multidirectional differentiation potential. Adipose tissue-derived mesenchymal stem cells (AT-MSCs) and BM-MSCs have liver differentiation potential in vivo and in vitro to acquire hepatocyte-like cell morphology and hepatocyte-specific markers (including albumin and alpha-fetoprotein) [86, 87]. Therefore, MSCs have the ability to differentiate into hepatocyte-like cells and are a promising source of liver regeneration [88, 89]. However, hepatic differentiation of MSCs is still insufficient for clinical application. They cannot effectively differentiate into liver cells but can be transformed into myofibroblasts, limiting their applications. Further research is needed to improve the efficacy and consistency of differentiation from MSCs to liver cells. After incubation with some specific growth factors, including hepatocyte growth factor (HGF) and basic fibroblast growth factor (bFGF), MSCs show high liver differentiation capacity [90]. Compared with undifferentiated cells, predifferentiation of AT-MSCs into hepatocytes in vitro contributes to a long-term liver function integration in vivo [91]. Compared with BM-MSCs cultured on uncoated plates, the viability and hepatocyte differentiation of BM-MSCs cultured on Matrigel and ECM coatings were significantly enhanced [92]. Engineered multicellular aggregates constructed from E-cadherin-modified microparticles have constructed a bionic microenvironment to promote endoderm differentiation and subsequent liver differentiation in human MSCs [93]. At the posttranscriptional level, microRNA is a key factor in cell differentiation and proliferation. Zhou et al. screened the best miRNA candidates for hepatocyte differentiation. MiR-30a and miR-1290 are essential for liver differentiation. The remaining five miRNAs (miR-122, miR-148a, miR-424, miR-542-5p, and miR-1246) are also crucial for this process [94]. Therefore, we can improve MSC hepatocyte-like cell differentiation ability by adding cytokines and growth factors, adjusting the microenvironment, modifying genes, and so on.

### 4.2. Paracrine Effect of Mesenchymal Stem Cells

Although MSCs have the ability to differentiate into hepatocyte-like cells, many studies have shown that other effects of MSCs in treating liver fibrosis can be attributed to paracrine effects [95, 96]. This secretion is due to the release of EVs and other soluble molecules by MSCs. Conditioned medium (CM) obtained from cultured MSCs contains a combination of EVs and soluble proteins. Both MSCs and MSC-CM can play a role in the pathogenesis of chronic fibrosis by acting on various key cells [97]. The therapeutic potential and mechanism of MSC-EVs have been described in detail above, and soluble molecules secreted by MSCs also have important roles. These paracrine factors, such as cytokines, growth factors, and chemokines, have effects including apoptosis, anti-inflammatory property, angiogenesis promotion, and repair [98].

Activation of HSCs has been recognized as the main driver of experimental liver fibrosis [99]. Transforming growth factor beta subtype 3 (TGF-*β*3) and hepatocyte growth factor (HGF) can induce G (0)/G (1) block in HSCs, thereby ameliorating liver fibrosis [100]. With the participation of the transcription factors nuclear factor *κ*B (NF-Kappa B) and B-cell leukemia-XL (Bcl-XL), NGF can enhance the apoptosis of HSCs [101]. Tumor necrosis factor-inducible gene 6 protein (TSG-6), a cytokine released from MSCs, influences MSC stemness and the biological effect of HSCs. Human primary HSCs treated with TSG-6 show significant downregulation of HSC activation markers and upregulation of senescence markers [102]. MFGE8 is an antifibrotic protein in the MSC secretome, which strongly inhibits TGF-*β* signaling and reduces extracellular matrix deposition and liver fibrosis in mice [103].

MSCs can show anti-inflammatory effects through increasing anti-inflammatory cytokines (interleukin (IL)-10, tumor necrosis factor-*α* (TNF-*α*)) and reducing proinflammatory cytokines (IL-1a, IL-6, IL-17, IFN- g, GCSF, GMCSF, MIP-2a, and MCP-1) [104, 105]. MSCs can secrete vascular endothelial growth factor (VEGF) and Ang1/Ang2 to promote angiogenesis. VEGF can also cooperate with HGF to stabilize the barrier function of endothelial cells [106]. Hepatocyte growth factor, fibroblast growth factor, insulin-like growth factor 1, and thymosin b4 (TB4) produced by MSCs have cytoprotective effects. In addition, VEGF-*α*, IGF-1, EGF, angiopoietin-1, matrix-derived factor-1, macrophage inflammatory protein-1 *α* and *β*, and erythropoietin are important molecules in normal wound healing [107].

Based on the important role of mesenchymal stem cells (MSCs) in the treatment of diseases, how to improve the efficacy of paracrine has attracted more and more attention. Previous studies have shown that MSC paracrine activity may vary with its microenvironment [108]. Recent experimental research results show that compared with conventional two-dimensional (2D) culture systems, three-dimensional (3D) culture systems (such as scaffolds, hydrogels, or spheres) enhance MSC paracrine secretion activity, promote the differentiation of MSCs, and maximize MSC potential for regeneration [82, 109, 110]. In addition, compared to MSCs with a single-gene modification and controlled MSCs, dually genetically modified MSCs have enhanced paracrine effects [111].

### 4.3. Immunoregulation of Mesenchymal Stem Cells

MSCs exert a wide range of immunosuppressive potentials and can regulate the activity of innate and adaptive immune system cells through cell-to-cell contact or secreted factors. However, the underlying mechanisms of MSC-mediated immune regulation have not been fully elucidated so far. Preliminary observations indicate that the immunomodulatory properties of MSCs derived from different sources are slightly different. For example, BM-MSCs and AT-MSCs have similar immunomodulatory capabilities [112, 113], but the difference in cytokine secretion leads to a stronger immunomodulatory effect in AT-MSCs than BM-MSCs. Adipose tissue-derived MSCs (AD-MSCs) express higher levels of IL-6 and transforming growth factor-*β* (TGF*β*) than bone marrow-derived MSCs (BM-MSCs). This is related to the higher metabolic activity of AD-MSCs [114]. However, it is unclear why MSCs from different tissues differ in their tissue-protective and immunomodulatory properties.

There is growing evidence that paracrine factors derived from MSCs can regulate the immune system by interacting with various immune cells, including macrophages, neutrophils, myeloid-derived suppressor cells, dendritic cells, natural killer (NK) cells, Kupffer cells, T lymphocytes, and B lymphocytes. Indoleamine-2,3-dioxygenase (IDO), and prostaglandin E2 (PGE2) secreted by MSCs are important paracrine factors for MSCs to exert immunosuppressive [115]. Specifically, IDO and PGE2 are key mediators of MSC-induced NK cell inhibition [116]. This inhibitory effect is associated with a sharp downregulation of the surface expression of activated NK receptors NKp30, NKp44, and NKG2D. IDO and PGE2 also inhibit the differentiation of Th1 cells, promote the differentiation of Tregs, and increase the migration of CD4 + T cells [117, 118]. IDO promotes the expansion of CD4 + FoxP3 + IL-10 + T regulatory cells and inhibits the proliferation of Th17 cells [119]. PGE2 is a soluble factor that mediates most of the immunosuppressive of Ad-MSCs and BM-MSCs on p-DC maturation and activates T lymphocyte proliferation [120]. Bone marrow dendritic cells (DCs) are also susceptible to this immunosuppressive [121]. The interaction between MSCs and Kupffer cells (KCs) has received little attention, but studies have shown that overexpression of PGE2 in MSCs increases the effect of MSCs on KC reprogramming [122]. In addition, UC-MSCs can increase IL-4 in vitro and in vivo and promote the mobilization of KCs, thereby reducing DMN-induced liver fibrosis [15].

Macrophages are one of the key cells connecting the innate and adaptive immune system. PGE2 secreted by MSCs plays a key role in manipulating the macrophage metabolic state and plasticity [123]. MSCs can cause macrophages to differentiate into immunosuppressive phenotypes and that these macrophages can inhibit T lymphocyte subsets at least as effectively as MSCs [124]. However, studies have shown that inhibition of M1 polarization during inflammation and inhibition of M2 polarization under anti-inflammatory conditions strongly depend on functional IL-6 signaling in macrophages. MSC-mediated macrophage polarization is strongly dependent on IL-6, while PGE2 has a smaller effect [125]. In MSC coculture and MSC-EV, due to the significant upregulation of PGE2 levels, the production of IL-23 and IL-22 is downregulated, thereby enhancing the anti-inflammatory phenotype of mature human regulatory macrophages (Mreg) [126].

In addition to interacting with various immune cells, PGE2 is able to induce MSC migration, which may be through the activation of EP2 receptors and FAK/ERK pathways to accelerate MSC homing efficiency [127]. The secretion of PGE2 can enhance the clearance of apoptotic cells (AC) by MSCs. Mechanistically, ACs stimulate MSCs to express COX2, thereby producing more PGE2 that suppresses T-cell responses. NF-*κ*B signaling pathway mediates COX2/PGE2 activation in MSCs [128].

MSCs are also capable of generating an immunoregulatory environment for Treg amplification through a variety of mechanisms [129]. MSCs induce the transformation of fully differentiated Th17 cells into functional Treg cells, thereby regulating the balance of Treg/Th17 cells in the CD4 + T cell population, which is partly attributed to HGF secreted by MSCs [130]. However, the number of MSCs and other cytokines also affect the Treg/Th17 ratio [131]. MSCs can inhibit the activation and differentiation of effector T cells by promoting the association of programmed death 1 (PD-1) with its ligands PD-L1 and PD-L2 [132, 133]. It is well established that nitric oxide (NO) production catalysed by iNOS leads to cell cycle arrest in T cells by affecting Janus kinase (JAK)—signal transduction and transcription activation (STAT) signaling pathways [134]. The lack of iNOS in MSCs also abolishes the therapeutic effects of MSCs in liver fibrosis models [135]. MSCs can also directly affect T cells by producing other immunosuppressive molecules, such as heme oxygenase 1 (HO1) [136], TGF*β* [137], and galectin [138, 139]. Compared with the extensive researches on the effect of MSCs on T cells, the effect of MSCs on B cell activation, proliferation, and B cell function has received less attention [140, 141].

### 4.4. Autophagy of Mesenchymal Stem Cells in Liver Fibrosis

Autophagy is a cell degradation pathway that uses lysosomes to degrade its damaged organelles and macromolecules. The amino acids and small molecules produced by it are reused to achieve intracellular material circulation and internal environment balance [142]. According to substrate degradation and substrate transport methods, autophagy can be categorized as macroautophagy, microautophagy, and molecular chaperone-mediated autophagy [143]. Generally, macroautophagy refers to autophagy, which is the most widely and clearly studied type [144]. In addition to its essential homeostasis, autophagy is involved in the development of many diseases, including cancer [145], inflammatory diseases [146], metabolic diseases [147], neurodegenerative diseases [148], and cardiovascular diseases [149]. Many studies have discussed the role of autophagy in these diseases and potential strategies for therapeutic regulation [150, 151]. In mesenchymal stem cells, autophagy also plays an important role in reducing inflammation, apoptosis, and oxidative stress in cells related to disease pathology and ultimately helps MSCs play a therapeutic role [152].

Autophagy affects the nature of MSCs and may have an impact on their regeneration and therapeutic potential. First, autophagy is closely related to aging [153]. Studies by Squillaro et al. have shown that MSCs in lysosomal storage disorders (LDS) are prone to apoptosis and aging due to impaired autophagy and DNA repair capabilities [154]. Zhang et al. observed that autophagy plays a protective role in D-gal-induced MSC aging, and ROS/JNK/p38 signaling plays an important mediating role in autophagy delaying MSC senescence [155]. In addition, reducing autophagy reduces hypoxia tolerance in aging MSCs [156].

Autophagy can regulate MSC-mediated immune regulatory functions, reduce inflammation, and promote anti-inflammatory effects. Cen et al. pretreated MSCs with 3-methyladenine (3-MA) and rapamycin to regulate autophagy and then cocultured them with CD4 + T cells [157]. The results showed that 3-MA inhibited autophagy in MSCs, while rapamycin was able to activate autophagy. Rapamycin increased the migration of CD4 + T cells, while 3-MA reduced their migration. They demonstrated that MSC autophagy increases the migration of CD4 + T cells through CXCL8 and promotes Treg cell differentiation, while suppressing Th1 cell differentiation by secreting TGF-*β*1. Similarly, Gao et al. believed that autophagy levels regulate the immune suppression of CD4 + T cells by MSCs by affecting the secretion of TGF-*β*1 [158]. The homeostasis of CD4 + T cells is considered to be the key in the process of hepatitis and liver fibrosis [159, 160].

Previous studies have shown that autophagy inhibition results in enhanced antifibrotic effect of MSCs. Wang et al. found that autophagy inhibition via Becn1 downregulation improves the mesenchymal stem cells antifibrotic potential in CCl4-induced mouse liver fibrosis model, which may be partially regulated by elevated PTGS2/PGE2 in the paracrine pathway [161]. Dang et al. found that autophagy promotes the apoptosis of MSCs induced by TNF-*α* and IFN-*γ*, and this effect may be related to the inhibition of autophagy in MSCs that upregulates PGE2 secretion [162]. Therefore, manipulation of autophagy in MSCs may be a new strategy to improve its antifibrotic ability.

## 5. Clinical Practice

### 5.1. Recently Started or Planned Clinical Trials

**Figure 3:**
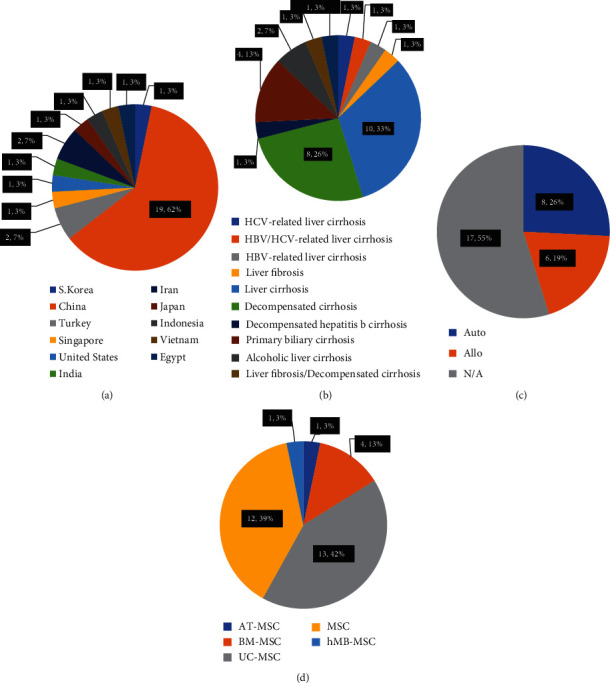
Recent trends in clinical trials using MSCs. Proportion of country (a), disease conditions (b), autologous or allogeneic (c), and sources of MSCs (d) in recent clinical trials.

A number of clinical trials have begun to evaluate the safety of MSCs in the treatment of patients with liver cirrhosis, observe the efficacy of MSCs treatment, and explore the optimal MSC infusion protocol. According to ClinicalTrials.gov, we evaluated clinical studies which began after 1 January, 2007. The results show that 31 clinical trials are registered. As shown in Table 1, 19 of the 31 cases (61.3%) are from China, 2 cases (6.5%) from Turkey, and 1 case (3.2%) occurs in S. Korea, Iran, Japan, Indonesia, Egypt, Vietnam, Singapore, the United States, and India (Figure 3(a)). The etiology of liver cirrhosis is slightly different in each of the 31 cases. 10 cases (32.3%) are liver cirrhosis, and 8 cases (25.8%) are decompensated cirrhosis. The third liver cirrhosis is primary biliary cirrhosis, which has 4 cases (12.9%). Alcoholic liver cirrhosis has 2 cases (6.5%), while HCV-related liver cirrhosis, HBV-related liver cirrhosis, liver fibrosis, and decompensated hepatitis b cirrhosis each has 1 case (3.2%). Liver fibrosis and decompensated cirrhosis result in 1 case (3.2%) while liver fibrosis and liver cirrhosis result in another. The etiology of liver cirrhosis manifests the morbidity of different diseases, and it may provide potential methods for corresponding intervention (Figure 3(b)). The source of MSCs also differs in 31 cases, 6 of the 31 cases (19.4%) were treated with allogeneic MSCs, and 8 of the 31 (25.8%) cases were treated with autologous MSCs (Figure 3(c)). Of the 31 cases, 17 cases (54.8%) were unclear whether allogeneic or autologous cells were used. However, the results still indicate an increase in the frequency of treatment with allogeneic MSCs compared to previously reported frequencies. In terms of cell origin, MSCs can be derived from umbilical cord tissue (13 cases, 41.9%), bone marrow (4 cases; 12.9%), adipose tissue (1 case; 3.2%), menstrual blood (1 case; 3.2%), and unknown (12 cases, 38.7%) (Figure 3(d)). The number of cells used in the experiment was slightly different, approximately 2-4 injections at a dose of 0.1–1.5 × 10^6^ cells/kg. Of the 31 cases, 9 (29.0%) were administered via the peripheral vein, 6 (19.4%) were administered via the hepatic artery, 1 (3.2%) were administered via the peripheral vein and hepatic artery simultaneously, and 15 were unknown (48.4%). Peripheral vein and hepatic arterial injections have become the main methods of cell administration. In addition, 2 of the 31 cases are more special. One is the safety study of the combined use of autologous CD34-selected cells and MSCs; the other is the safety study of the combined use of MSCs and Tregs, suggesting that combined cell therapy may become an effective treatment. Most of the 31 clinical trials are still in phase I or/and II, and one of them is in phase IV clinical trials.

### 5.2. Published Reports on the Results of Clinical Trials Conducted by MSCs

The above shows 31 completed or ongoing clinical trials of MSC therapy or MSC combined cell therapy for liver cirrhosis (LC). At the same time, many reports have been published describing the results of clinical trials using MSCs. Sang et al. published a meta-analysis to evaluate the efficacy and safety of UMSCs combined with traditional supportive therapy (TST) in the treatment of LC patients [163]. They evaluated a total of 14 trials including 717 LC patients who met their selection criteria. The results of their meta-analysis indicated that the combination of UMSCs and TST had more satisfied therapeutic effects for LC patients than TST alone (improved liver function is shown by reduced total bilirubin, alanine aminotransferase, prothrombin time, increased serum albumin, cholinesterase, and prothrombin activity).

Jang et al. reported a phase II study whose purpose was to elucidate the antifibrotic effect of BM-MSCs in patients with alcoholic cirrhosis [164]. Twelve baseline patients with alcoholic liver cirrhosis confirmed by alcohol biopsy were recruited, BM-MSCs were isolated from each patient's bone marrow and expanded for 1 month. Then, 5 × 10^7^ cells were injected twice through the hepatic artery at 4 and 8 weeks. Finally, laboratory examination and biopsy results 12 weeks after the second injection showed that histological improvement was observed in 6 (54.5%) of the 11 patients (one patient withdrew because of drinking). Ten patients' Child-Pugh scores (90.9%) had been improved. In addition, Suk et al. subsequently reported a multicenter, randomized, open-label, phase 2 trial to evaluate the effectiveness and safety of autologous BM-MSC transplantation in the treatment of alcoholic cirrhosis [165]. The results showed that fibrotic areas were reduced, and liver function was significantly improved. Other studies have shown that MSC is a potential choice for the treatment of liver cirrhosis caused by autoimmune diseases [166], and it also plays a therapeutic role in decompensated cirrhosis [167].

## 6. Conclusion

Liver fibrosis is a pathophysiological process caused by various pathogenic factors and abnormal hyperplasia of connective tissue in liver. If the pathogenic factors cannot be eliminated in time, the process of fibrosis will continue for a long time and develop into cirrhosis. At present, there is no effective treatment for liver cirrhosis. In recent years, many experimental studies and clinical trials have shown that MSCs have a good therapeutic effect in the treatment of liver fibrosis and cirrhosis. Especially, many new therapies, such as MSC sheets, MSCs-EV, gene-modified MSCs, and MSC spheroids, have significantly improved the effect of MSCs in the treatment of liver fibrosis. Although some products of MSCs are approved to be listed in some countries, their safety and effectiveness have not been officially recognized. At present, there is no standardized treatment strategies for MSCs, including the source of MSCs, treatment indications, optimal dosage, administration time, and delivery route. Therefore, it is difficult to apply MSC-based liver fibrosis therapy to clinical practice in the short term. Nevertheless, we believed that cell-free therapy based on MSCs, gene-modified or pretreated MSCs, and MSC spheroids may be the research hotspot and development trend in the future. In conclusion, MSC-based therapies have great potential for the alleviation or treatment of liver fibrosis. However, the challenges associated with this therapy must be addressed before it can be widely used in clinical practice. Therefore, MSC-based therapy still needs to be further studied before widely clinical application.

## Figures and Tables

**Table 1:** Summary of clinical trials using mesenchymal stem cells for the treatment of liver fibrosis.

ClinicalTrials.gov identifier	Country	Conditions	Phase	Estimated enrollment	Allocation/masking	Intervention model	Biological	Auto/Allo	Cell number	Cell injection times	Cell injection route
NCT02705742	Turkey	HCV-related liver cirrhosis	I/II	5 participants	N/A/open label	Single group assignment	AT-MSC	Auto	1 × 10^6^/kg	N/A	Hepatic artery and peripheral vein
NCT02652351	China	Liver cirrhosis	I	20 participants	N/A/open label	Single group assignment	UC-MSC	Allo	A single dose of 2 × 10^7^	4	Intravenous or hepatic artery
NCT02786017	China	Decompensated cirrhosis	I/II	40 participants	Randomized/double (participant, outcome assessor)	Parallel assignment	Injectable Collagen scaffold + HUC-MSCs	Allo	The total amount was 5 × 10^8^	N/A	peripheral vein
NCT03209986	China	HBV/HCV-related liver cirrhosis	N/A	200 participants	Randomized/single (outcomes assessor)	Parallel assignment	MSC	N/A	1 × 10^6^/kg	3	Peripheral vein
NCT03668145	China	Primary biliary cirrhosis	N/A	140 participants	Randomized/quadruple (participant, care provider, investigator, outcome assessor)	Parallel assignment	MSC	N/A	0.1 − 1 × 10^6^/kg	3	Peripheral vein
NCT03529136	China	Decompensated cirrhosis	II	252 participants	Nonrandomized/open label	Parallel assignment	UC-MSC	Allo	1.5 × 10^6^/kg	2~4	Peripheral vein
NCT03626090	Singapore	Liver cirrhosis	I/II	20 participants	N/A/open label	Single-group assignment	BM-MSC	Auto	0.5 − 1 × 10^6^/kg	N/A	Peripheral vein
NCT03826433	China	Decompensated hepatitis b cirrhosis	I	20 participants	Nonrandomized/open label	Parallel assignment	UC-MSC	Allo	6 × 10^7^/30 ml	N/A	Peripheral vein
NCT03945487	China	Decompensated cirrhosis	II	200 participants	Randomized/open label	Parallel assignment	UC-MSC	Allo	1.0 × 10^6^/kg	3	Intravenous
NCT03838250	United States	Alcoholic liver cirrhosis	I	10 participants	N/A/open label	Single-group assignment	Cellgram™ (BM-MSC)	Auto	5 × 10^7^/10 ml	N/A	Hepatic artery
NCT04243681	India	Decompensated cirrhosis	IV	5 participants	Nonrandomized/open label	Parallel assignment	CD 34 and MSC	Auto	N/A	N/A	Hepatic artery
NCT03460795	China	Decompensated cirrhosis	I/II	30 participants	N/A/open label	Single-group assignment	MSCs and Tregs	N/A	N/A	N/A	N/A
NCT01454336	Iran	Liver fibrosis/decompensated cirrhosis	I	3 participants	N/A/open label	Single-group assignment	MSC	Auto	N/A	N/A	N/A
NCT01220492	China	Liver fibrosis	I/II	266 participants	Randomized/open label	Parallel assignment	UC-MSC	N/A	N/A	N/A	N/A
NCT01342250	China	Decompensated cirrhosis	I/II	20 participants	Randomized/open label	Parallel assignment	UC-MSC	N/A	N/A	N/A	N/A
NCT01499459	Turkey	Liver cirrhosis	N/A	25 participants	N/A/open label	Single-group assignment	MSC	Auto	N/A	N/A	N/A
NCT01233102	China	Liver cirrhosis	I/II	200 participants	Randomized/single (single (participant))	Parallel assignment	MSC	N/A	N/A	N/A	Hepatic artery or vein
NCT01741090	Republic of Korea	Alcoholic liver cirrhosis	II	12 participants	N/A/open label	Single-group assignment	MSC	N/A	N/A	N/A	N/A
NCT00976287	China	Liver cirrhosis	II	50 participants	Randomized/single (single (participant))	Parallel assignment	BM-MSC	Auto	N/A	N/A	Hepatic artery
NCT00993941	China	Liver cirrhosis	II	60 participants	Nonrandomized/open label	Parallel assignment	BM-MSC	N/A	N/A	N/A	N/A
NCT01728727	China	HBV-related liver cirrhosis	I/II	240 participants	Randomized/open label	Parallel assignment	UC-MSC	N/A	N/A	N/A	N/A
NCT01483248	China	Liver fibrosis/liver cirrhosis	I/II	50 participants	Randomized/open label	Single-group assignment	hMB-MSC	N/A	N/A	N/A	N/A
NCT03254758	Japan	Decompensated cirrhosis	I/II	15 participants	N/A/open label	Single-group assignment	MSC	N/A	N/A	N/A	N/A
NCT04357600	Indonesia	Liver cirrhosis	I/II	12 participants	N/A/open label	Single-group assignment	UC-MSC	N/A	N/A	N/A	N/A
NCT01573923	China	Liver cirrhosis	I/II	320 participants	Nonrandomized/open label	Parallel assignment	UC-MSC	N/A	N/A	N/A	N/A
NCT01224327	China	Liver cirrhosis	I/II	50 participants	Randomized/open label	Parallel assignment	UC-MSC	N/A	N/A	N/A	Hepatic artery
NCT04522869	Vietnam	Primary Biliary cirrhosis	I/II	34 participants	Nonrandomized/open label	Parallel assignment	UC-MSC	N/A	N/A	N/A	N/A
NCT01662973	China	Primary biliary cirrhosis	I/II	100 participants	Randomized/open label	Parallel assignment	UC-MSC	N/A	N/A	N/A	N/A
NCT02943889	Egypt	Liver cirrhosis	I/II	40 participants	Nonrandomized/open label	Parallel assignment	MSC	N/A	N/A	N/A	N/A
NCT00476060	Iran	Decompensated cirrhosis	II	36 participants	Randomized/double (participant, outcome assessor)	Parallel assignment	MSC	Auto	N/A	N/A	N/A
NCT01440309	China	Primary biliary cirrhosis	I	20 participants	Randomized/open label	Parallel assignment	MSC	Allo	N/A	N/A	N/A

## Data Availability

The references used to support the findings of this study are included within the article.
